# The Cold Morning Bath

**Published:** 1899-10

**Authors:** 


The cold morning bath, far from being always beneficial, is distinctly injurious after a certain age, we are told by *The Hospital*. Says this journal: "Many people who have, as they would say, been 'always accustomed' to take a cold tub every morning continue the habit long after it had better have been given up. They do this partly because it is a habit, and partly because they dislike the confession of getting old which seems to be involved in giving up the customs of their more youthful days. But we are quite clear that unless good reaction very quickly follows a cold bath, and follows it without much 'toweling,' such tubbing is very often injurious. Whenever a man has to 'rub himself warm,' or when he finds that he is not right again until after his breakfast, he may feel sure that his tub is doing him harm, and that he would do better to take a warm bath, finishing off with a rapid sponge over with cold water."

Examining Michigan Recruits.—"Don't this old injury hurt you when you attempt to run?" asked the examining surgeon of a candidate for enlistment. "Course it does. If yer looking for soldiers what's goin' to run, just count me out."—*Detroit Free Press.*

